# Hexagonal-structured *ε-*NbN: ultra-incompressibility, high shear rigidity, and a possible hard superconducting material

**DOI:** 10.1038/srep10811

**Published:** 2015-06-01

**Authors:** Yongtao Zou, Xuebing Wang, Ting Chen, Xuefei Li, Xintong Qi, David Welch, Pinwen Zhu, Bingbing Liu, Tian Cui, Baosheng Li

**Affiliations:** 1 Mineral Physics Institute, State University of New York, Stony Brook, N.Y. 11794, United States; 2 State Key Laboratory of Superhard Materials, College of Physics, Jilin University, Changchun, 130012, China; 3 Department of Geosciences, State University of New York, Stony Brook, N.Y. 11794, United States; 4 Department of Materials Science and Engineering, State University of New York, N.Y. 11794, United States; 5 Condensed Matter Physics and Materials Science Department, Brookhaven National Laboratory, Upton, N.Y. 11973, United States

## Abstract

Exploring the structural stability and elasticity of hexagonal *ε*-NbN helps discover correlations among its physical properties for scientific and technological applications. Here, for the first time, we measured the ultra-incompressibility and high shear rigidity of polycrystalline hexagonal *ε*-NbN using ultrasonic interferometry and *in situ* X-ray diffraction, complemented with first-principles density-functional theory calculations up to 30 GPa in pressure. Using a finite strain equation of state approach, the elastic bulk and shear moduli, as well as their pressure dependences are derived from the measured velocities and densities, yielding *B*_*S0*_ = 373.3(15) GPa, *G*_*0*_ = 200.5(8) GPa, ∂*B*_*S*_*/∂P* = 3.81(3) and ∂*G*/∂*P* = 1.67(1). The hexagonal *ε*-NbN possesses a very high bulk modulus, rivaling that of superhard material *c*BN (*B*_*0*_ = 381.1 GPa). The high shear rigidity is comparable to that for superhard *γ*-B (*G*_*0*_ = 227.2 GPa). We found that the crystal structure of transition-metal nitrides and the outmost electrons of the corresponding metals may dominate their pressure dependences in bulk and shear moduli. In addition, the elastic moduli, Vickers hardness, Debye temperature, melting temperature and a possible superconductivity of hexagonal *ε*-NbN all increase with pressures, suggesting its exceptional suitability for applications under extreme conditions.

Transition-metal nitrides have recently attracted considerable interest in condensed matter physics, solid-state chemistry and materials science because of their unique/superior physical properties such as low compressibility, high hardness, excellent electronic and magnetic properties, as well as the superconductivity with relatively high transition temperature for scientific and technological applications^1^,^2^,^3^,^4^,^5^,^6^,^7^. These nitrides are usually metallic and highly refractory, which exhibit more excellent properties such as hardness and elasticity than the corresponding transition-metals themselves^1^,^2^. Among these nitrides, cubic *δ*-NbN, due to its exceptional mechanical and superconducting properties, is particularly attractive to scientists and is considered as a promising material used in carbon nanotube junctions^8^, radio frequency superconducting accelerator cavities^9^, hot electron photodetectors^10^, and so on.

Elastic bulk and shear moduli as well as their pressure dependences are important parameters in understanding the structural behavior and physical/mechanical properties of materials. The crystal structures of the transition-metal nitrides are generally characterized by strong intermetallic bonding with transition-metal atoms and N atoms occupying octahedral, tetrahedral or trigonal prismatic sites, giving rise to a large cohesive energy. It is known that NbN possesses many polymorphs^3^,^4^,^11^,^12^,^13^,^14^,^15^,^16^, but only cubic *δ*-NbN has been extensively investigated by different experimental techniques^3^,^4^ and theoretical calculations^11^,^12^,^15^,^16^,^17^,^18^. Hardness measurements using the Vickers micro-indentation method were performed to study the mechanical properties of *δ*-NbN, yielding a Vickers hardness of 17~20 GPa as same as that of sapphire (18~20 GPa)^4^. Static compression experiments^4^ gave a bulk modulus of *δ*-NbN (*B*_*T0 *_= 354 GPa), which is comparable to that of superhard material *c*BN (381.1 GPa)^19^. Recently, Wang *et al.*^12^ reported first-principles calculations of the elastic constants, thermodynamic properties and structural phase transitions of NbN polymorphs (*i.e.* NaCl-type, NiAs-type and WC-type NbN) under high pressure. The phonon and total-energy calculations by Wang *et al.*^12^ showed that the cubic *δ*-NbN was metastable and the hexagonal-structured NbN (*e.g.* WC-type structure) was more stable than the cubic counterpart. In addition, it was predicted that a hexagonal-structured NbN exhibits higher hardness, bulk and shear moduli compared to those for cubic *δ*-NbN^13^. For hexagonal *ε*-NbN polymorph, despite its crystal structure was discussed by Terao^14^, experimental studies on the elastic/mechanical properties of *ε*-NbN have never been reported, in particular for the shear related properties which are important quantities for technological and engineering applications. Here, for the first time, we report the high shear rigidity and ultra-incompressibility of polycrystalline hexagonal structured *ε*-NbN studied by using ultrasonic measurements in a multi-anvil apparatus and *in situ* synchrotron X-ray diffraction in a diamond-anvil-cell (DAC), in conjunction with first-principles density functional theory calculations using the local density approximation (LDA).

## Results

**Figure 1:**
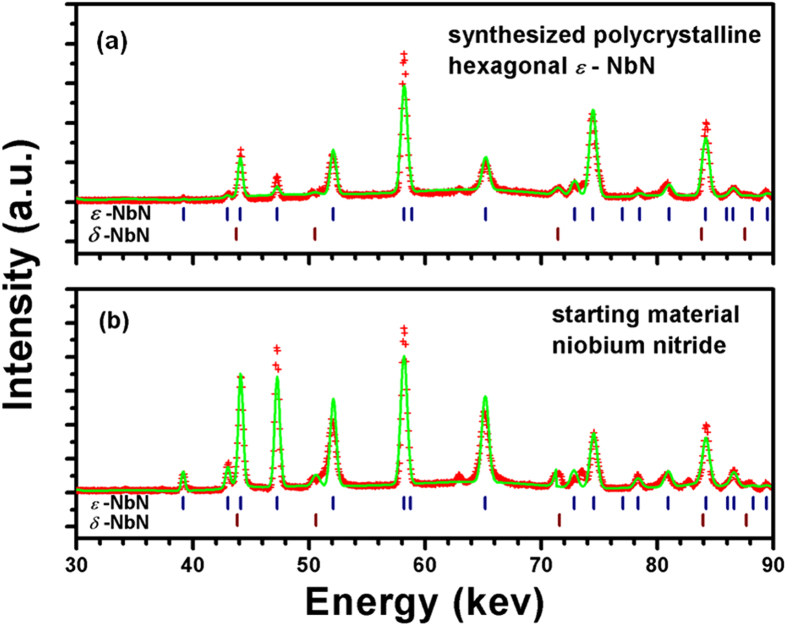
Synchrotron *in situ* X-ray diffraction pattern of the high-pressure synthesized bulk polycrystalline niobium nitride for the current ultrasonic measurements (**a**), suggesting that the synthesized specimen is a hexagonal structured *ε*-NbN (space group: *P6*_*3*_*/mmc*, No. 194), in comparison with that of NbN starting material (**b**). Red crosses and green lines denote the observed and calculated profiles, respectively. The tick marks correspond to the peak positions of the hexagonal *ε*-NbN (PDF: #89-4757) and cubic *δ*-NbN (PDF: #74-1218).

Bulk polycrystalline hexagonal *ε*-NbN specimens used for the present sound velocity measurements were prepared at high pressure and high temperature. *In situ* ultrasonic measurements on *ε*-NbN were performed at pressures up to ~12 GPa in a multi-anvil high-pressure apparatus. The experimental procedure in details can be found in the “Methods” section. Figure. 1a shows an X-ray diffraction pattern of the synthesized bulk specimen used for the current acoustic measurements. For comparison, the corresponding X-ray diffraction of the niobium nitride powder starting material (as purchased from Goodfellow) is also shown in Fig. 1b, indicating that the synthetic specimen is almost a pure phase of *ε*-NbN with the hexagonal structure (PDF: #89-4757) coexisting with a minor amount of cubic *δ*-NbN. The volume fraction of cubic *δ*-NbN was estimated to be ~1% from the intensity of the *δ*-NbN peaks observed in X-ray diffraction. Using the Voigt bound for our calculation^20^, the abundance of cubic *δ*-NbN of ~1% will result in less than 1% difference in elastic moduli as compared with those for pure hexagonal-structure *ε*-NbN. The difference is within the current measurement uncertainties, indicating the effect of the minor cubic *δ*-NbN on the elasticity of synthesized nominal hexagonal-structured *ε*-NbN can be negligible.

**Figure 2:**
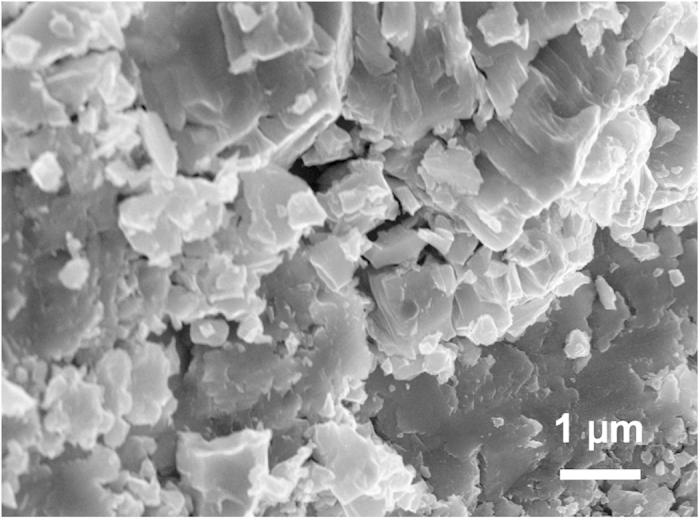
SEM image showing the microstructure of the synthesized polycrystalline hexagonal-structured *ε*-NbN for the present sound velocity measurements. The synthetic specimen was free of visible microcracks with an average grain size of about 1-2 *μ*m, exhibiting an equilibrated microstructure with homogeneous fine grains.

As shown in Fig. 2, a representative SEM image revealed that the synthetic specimen was free of visible microcracks with an average grain size of about 1-2 *μ*m, and exhibited an equilibrated microstructure with homogeneous fine grains. Further composition analyses of the synthesized specimen yield Nb_0.98(2)_N_0.96(5)_O_0.06(4)_ as determined by the SEM-EDX measurements, indicating that the high-pressure synthesized specimen is almost oxygen-free *ε*-NbN or stoichiometric nitride within its uncertainty. As measured by Archimedes immersion method, the bulk density of the sample was determined to be 8.30(2) g/cm^3^ with a porosity of ~0.5%.

Using the initial sample length (*L*_*0*_), zero-pressure density (*ρ*_*0*_), and travel times (*t*_*p*_ and *t*_*s*_) at high pressures, we determine the sample lengths using Cook’s method^21^ described as 
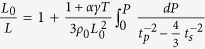
, where *L* is sample length, *γ* is the Grüneisen parameter, *α* is the thermal expansivity, and *T* is temperature. To date, no thermal expansion coefficient (*α*) and Grüneisen parameter (*γ*) data of hexagonal *ε*-NbN are available, so we take *α* ≈ 2 × 10^−5^ K^−1^ and *γ* ≈ 2.00 for the calculation of the sample length (*L*), yielding the value of *αγT* for *δ*-NbN equal to ~0.009 at room temperature Actually, for a wide range of materials^20^,^22^ at room temperature (*T*~300 K), the values of thermal expansion coefficient (*α*) and Grüneisen parameter (*γ*) are found in the range of *α* *=* 0.5~3 × 10^−5^ K and *γ* *=* 1~2 , so the value of *αγT* ranges from 0.0 to 0.02 and is often assumed to be ~0.01 (Ref. 20).This range of *αγT* introduces an error of ± 0.02% in the resultant length which is well within the claimed uncertainty in Table 1. Since no length/diameter change occurs after our acoustic experiments, it is reasonable to assume that the compression is purely elastic, and thus the densities of the sample under pressure can be determined from the length changes 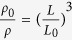. The derived sample length (*L*), elastic wave velocities (*V*_*P*_ and *V*_*S*_), elastic bulk and shear moduli (*B*_*S*_, *G*) and Poisson’s ratio (*v*) are shown in Table 1.

**Figure 3:**
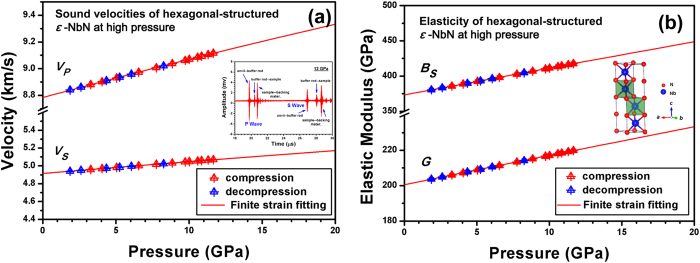
**(a)** Elastic wave velocities (*V*_*P*_ and *V*_*S*_) of polycrystalline hexagonal *ε*-NbN at high pressure. A representative acoustic echoes of the current ultrasonic measurements at the highest pressure of ~12 GPa is shown as an inset. (**b**). Elastic bulk and shear moduli (*B*_*S*_ and *G*) of polycrystalline hexagonal *ε*-NbN at high pressure. Insets in red circles denote velocities upon compression, and blue solid circles symbols those during decompression. Red solid lines are from the third-order finite strain fits.

Figure. 3a shows the elastic wave velocities (*V*_*P*_ and *V*_*S*_) of hexagonal *ε*-NbN during compression as well as decompression as a function of pressure. Fitting the present sound velocity data to the third-order finite strain equations^22^,^23^, we obtained compressional (*V*_*P*_ = 8.77(1) km/s) and shear (*V*_*S*_ = 4.91(1) km/s) wave velocities at ambient conditions, which are in good agreement with the results from our first-principles calculations (*V*_*P*_ = 8.5 km/s and *V*_*S*_ = 4.9 km/s). It is found that both compressional and shear velocities increase monotonically with increasing pressure. Clearly, the sound velocities and elastic moduli of *ε*-NbN during compression and those obtained on decompression exhibit an exceptional consistency, suggesting that non-hydrostatic stresses in the current ultrasonic measurements are negligible. The elastic bulk (*ρV*_*P*_^*2*^ *=* *B*_*S*_ *+ 4G/3*) and shear (*G* *=* *ρV*_*S*_^2^) moduli calculated from *P* and *S* wave velocities and densities are given in Table 1. As seen from Fig. 3b, the elastic bulk modulus exhibits a linear increase with increasing pressure and reaches ~450 GPa at a pressure of 20 GPa, almost the same as the zero-pressure value of the bulk modulus for diamond (~446 GPa)^24^. The shear modulus/rigidity also increases within the whole pressure range, and is equal to ~234 GPa at pressures around 20 GPa.

To obtain the zero-pressure adiabatic bulk and shear moduli, as well as their pressure dependences, the velocity and density data can be fitted simultaneously to the finite strain equations (Eqs. 1 and 2) without the explicit input of pressure^25^,
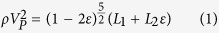

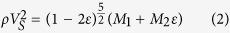
in which *M*_*1*_ = *G*_*0*_*, M*_*2*_ = 5*G*_*0*_ -3*B*_*S0*_
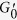
*, L*_*1*_ = *B*_*S0*_ + 4*G*_*0*_*/*3, and *L*_*2*_ = 5*L*_*1*_ *–* 3*B*_*S0*_ (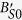 + 4
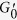
*/*3). The strain ε is defined as 
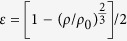
. The fitted coefficients, *L*_*1*_*, L*_*2*_*, M*_*1*_, and *M*_*2*_, obtained by minimizing the difference between the calculated and the observed compressional and shear wave velocities, are used for the calculation of the zero-pressure adiabatic bulk (*B*_*S0*_) and shear (*G*_*0*_) moduli, as well as their pressure derivatives (*әB*_*S*_*/әP* and *әG/әP*). Applying the above fitting procedures to the current velocity and density data yield *B*_*S0*_ = 373.3(15) GPa, *G*_*0*_ = 200.5(8) GPa, *әB*_*S*_*/әP* = 3.81(3), and *әG/әP* = 1.67(1); the total root-mean-square misfits for *B*_*S*_ and *G* are about 0.08 and 0.03 GPa, respectively (Fig. 3b).

## Discussion

The elastic properties obtained from our ultrasonic measurements and first-principles calculations are shown in Table 2. The experimentally obtained bulk modulus *B*_*S0*_ = 373.3 GPa is comparable to our theoretical Voigt bulk modulus (*B*_*V *_= 355 GPa), whereas the shear rigidity exhibits almost the same value as that from our first-principles calculations (Table 2). In comparison with cubic *δ*-NbN, the hexagonal *ε*-NbN is much less compressible, and shows a higher shear resistance. For a better comparison, we have summarized the physical properties of *ε*-NbN together with those of other transition-metal nitrides and superhard materials in Table 3. As indicated by the elastic bulk modulus (the inverse of compressibility, or incompressibility), hexagonal *ε*-NbN (*B*_*S0*_ = 373.3 GPa) is as incompressible as hexagonal *δ*_*3*_-MoN (379.4 GPa) and superhard *c*BN (381.1 GPa), as well as the nobel metal nitride PtN (~372 GPa), but slightly more compressible than the WC-type TaN (398 GPa), and less compressible than the WC-type NbN (357 GPa)^26^, VN (336 GPa)^26^, and cubic *δ*-NbN which exhibits a scattered bulk modulus ranging from 292 to 354 GPa owing to different specimens prepared by various experimental techniques, as well as different approximations in theoretical calculations^4^,^17^,^18^. It is found that the bulk modulus for *ε*-NbN is more than ~43% higher than that for the superhard *γ*-B (213.9 GPa)^27^. In contrast, the high shear rigidity (*G*_*0*_ = 200.5 GPa) for *ε*-NbN is comparable to that for superhard *γ*-B (*G*_*0*_ = 227.2 GPa)^27^, as well as those for WC-type NbN (*G*_*0*_ = 226 GPa) and VN (*G*_*0*_ = 220 GPa) from the previous theoretical calculations^26^. Our experimentally obtained shear rigidity is significantly lower than those for the hexagonal *δ*_*3*_-MoN (*G*_*0*_ = 248.2 GPa)^28^ and *c*BN (*G*_*0*_ = 398.8 GPa)^19^, but larger than that for cubic *δ*-NbN (*G*_*0*_ = 161 GPa)^4^.

As shown in Table 3, the pressure dependence of the bulk modulus (*B’* = 3.81) from the present experimental study is almost as same as the previous theoretical results for WC-type structured TaN (*B’* = 3.83), NbN (*B’* = 3.89) and VN (*B’* = 3.82), but significantly smaller than that for *δ*_*3*_-MoN (*B’* = 4.44). The almost same pressure dependences of bulk modulus (~3.8-3.9) in hexagonal WC-type TaN, NbN and VN, as well as the hexagonal *ε*-NbN are observed, indicating that this behavior is mainly dominated by its crystal structure and the same outmost electrons of the transition metals such as V, Nb and Ta. If this is also applicable for the shear rigidity, the corresponding *әG/әP* for the hexagonal WC-type TaN, NbN and VN, as well as *ε*-NbN will be expected to exhibit similar value as well. Our comparison shows that the hexagonal *ε*-NbN possesses superior mechanical/elastic properties, exhibiting very high bulk modulus, which can rival that of superhard material *c*BN (*B*_*0*_ = 381.1 GPa, Ref. 19). Its shear rigidity is comparable to that for superhard *γ*-B (*G*_*0*_ = 227.2 GPa, Ref. 27). The superior mechanical properties may originate from a particular σ-band of bonding states between the non-metal *p* orbitals and the metal *d* orbitals that strongly resists the shear strains^29^.

The Pugh modulus ratio *k* *=* *B/G*, namely the ratio between the bulk and shear moduli, is often used to predict the brittle or ductile behavior of materials. Based on the Pugh criterion^30^, materials having *B/G* > 1.75 exhibit ductile behavior; otherwise they behave in a brittle manner. As shown in Table 3, the Pugh modulus ratio *k* in the present work is 1.861, indicating that the hexagonal *ε*-NbN is a ductile material. Compared with cubic *δ*-NbN (*k* = 2.199), WC-type nitrides (TaN, NbN and VN), *δ*_3_-MoN (*k* = 1.529), superhard *γ*-B (*k* = 0.941) and *c*BN (*k* = 0.956), the hexagonal structured *ε*-NbN is more brittle than cubic *δ*-NbN, but more ductile than WC-type nitrides, superhard *γ*-B and *c*BN materials (Table 3). According to the elastic properties of *ε*-NbN (Table 3), the present Pugh modulus ratio *k* increases with increasing pressure, and reaches *k* = 1.92 at 20 GPa, indicating that the *ε*-NbN becomes even more ductile under high pressure.

**Figure 4:**
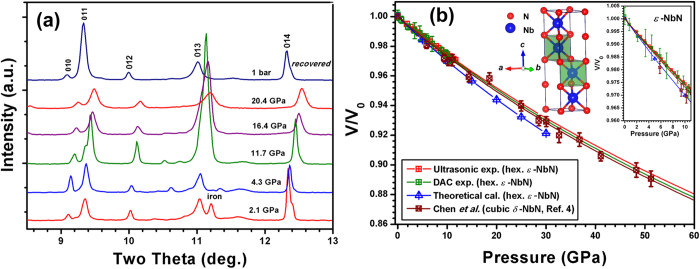
**(a)** Selected *in situ* synchrotron angle-dispersive X-ray diffraction patterns of hexagonal *ε*-NbN at high pressure. (**b**). Normalized unit-cell volumes as a function of pressure for hexagonal *ε*-NbN from the present static compression (DAC) and sound velocity measurement experiments, compared with the previous study on cubic *δ*-NbN by Chen *et al*^4^. Insets are the amplified *P-V* relations and crystal structure of hexagonal-structure *ε*-NbN. Crossed red squares and red curve symbolize the data points from the present ultrasonic measurements, and the related fitting results using the finite-strain equations^22^, respectively. Crossed green squares and green curve represent the data points as well as their fitting results using Birch-Murnaghan EOS from the present DAC measurements. Crossed brown squares are from the previous study on *δ*-NbN by Chen *et al*^4^. Crossed blue triangles are from our first-principles calculations.

*In situ* synchrotron X-ray diffraction patterns of hexagonal *ε-*NbN upon compression in a diamond-anvil cell (DAC) show that the hexagonal *ε-*NbN remains stable at pressures up to ~20 GPa (Fig. 4a). Figure. 4b shows the measured pressure-volume (*P-V*) relations or equation of state (EOS) for *ε*-NbN from the present DAC experiments, compared with those from our ultrasonic study and first-principles calculations, as well as the previous study on cubic *δ*-NbN^4^. The *P-V* data are fitted using a third-order Birch-Murnaghan equation of state^31^, yielding *B*_*T0 *_= 360(7) GPa and *V*_*0*_ = 85.80 (11) Å^3^ with *B*_*T*_’ = 3.8 (fixed). The obtained bulk modulus (*B*_*T0*_ = 360(7) GPa) is comparable to that (*B*_*S0*_ *=* 373.3 (15) GPa) from our ultrasonic measurements, and shows a slightly larger value versus that (*B*_*T0*_ = 354 GPa) for cubic *δ*-NbN^4^, as well as our theoretical results (355 GPa) (see Tables 2 and 3). These results from our studies of ultrasonic measurements (red curve) and DAC experiments (green curve), suggest that the hexagonal *ε-*NbN is less compressible than the cubic *δ*-NbN (brown curve) as reported by Chen *et al*^4^. In contrast, the current theoretical results (blue color) indicate that the hexagonal *ε*-NbN seems compressible than the cubic *δ*-NbN^4^. This difference might originate from the overbinding of LDA which yields lower *V/V*_*0*_ than experimental values at high pressures.

The experimental hardness of the hexagonal-structured *ε*-NbN was measured by means of a Vickers indentation method, yielding *H*_*V*_ = 21.5 GPa under the loading of 9.8 N which is in good agreement with our theoretical calculation results of ~18.5 GPa on the basis of the empirical hardness model^32^. Clearly, the hexagonal *ε*-NbN is almost as hard as cubic *δ*-NbN (17~20 GPa) and sapphire Al_2_O_3_ (21~23 GPa)^4^. Supposing that this hardness model^32^ is also applicable for materials under high pressure, a hardness as high as 30 GPa is predicted for *ε*-NbN at pressure of 200 GPa.

It is known that the acoustic modes of lattice vibration are related to the elastic wave velocities (*V*_*P*_ and *V*_*S*_). The acoustic Debye temperature (*Θ*) is described as 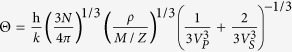 in which *M* is the molecular mass; Z is the number of atoms in the molecular formula; and *k, h, N* are Boltzmann’s constant, Planck’s constant, and Avogadro’s number, respectively. Using the experimentally determined elastic wave velocities and the density data at ambient condition (*V*_*P*_ = 8.79(2) km/s, *V*_*S*_ = 4.91(1) km/s, *ρ* = 8.30(1) g/cm^3^), the Debye temperature *Θ*_*0*_ is determined to be 738 K, which is almost the same as *Θ*_*0*_ *=* 737 K for NiAs-type NbN from theoretical calculations^16^ and is also comparable to *Θ*_*0*_ *=* 754 K for WC-type-structured NbN^16^.

**Figure 5:**
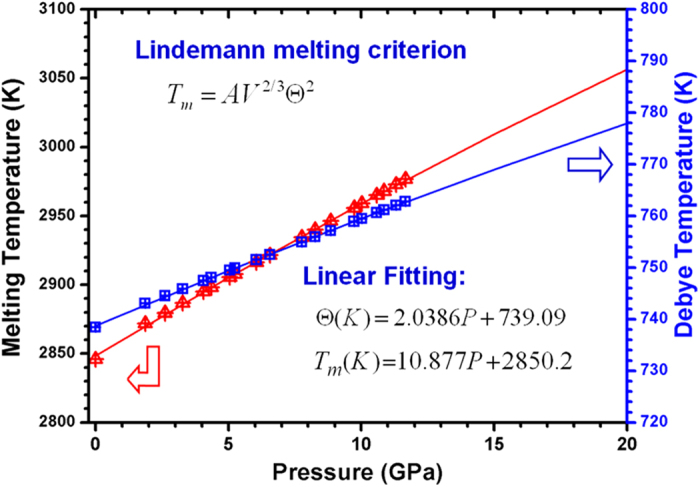
Calculated melting curve of hexagonal *ε*-NbN from the Lindemann melting criterion and the Debye temperature (*Θ*) as a function of pressure.

Using well-known thermodynamic methods, we can obtain the melting temperature (*T*_*m*_) variation at high pressure described as 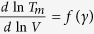, where *γ* is the thermodynamic Grüneisen parameter. According to the Gilvarry’s rule^33^, the best known representation of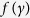 is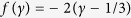. For a Debye solid, the thermodynamic Grüneisen parameter (*γ*) can also depicted as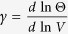. By combining the above two equations, *T*_*m*_ can be obtained: 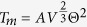, where *A* is a constant. This equation is exactly the Lindemann melting criterion^34^. Because of the lack of a value of the melting temperature (*T*_*m*_) of hexagonal *ε*-NbN at ambient pressure, the criterion point of *T*_*m*_ is thus taken from that of *δ*-NbN (*T*_*m*_ = 2846 K). By applying this model, the melting curve of hexagonal *ε*-NbN, together with the Debye temperature as a function of pressure, are shown in Fig. 5. Linear fittings of the Debye temperatures (*Θ*) and melting temperatures (*T*_*m*_) at high pressure, we obtain the equations 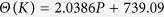 and 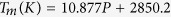, respectively. It is seen from Fig. 5 that both *Θ* and *T*_*m*_ increase monotonically with increasing pressure.

**Figure 6:**
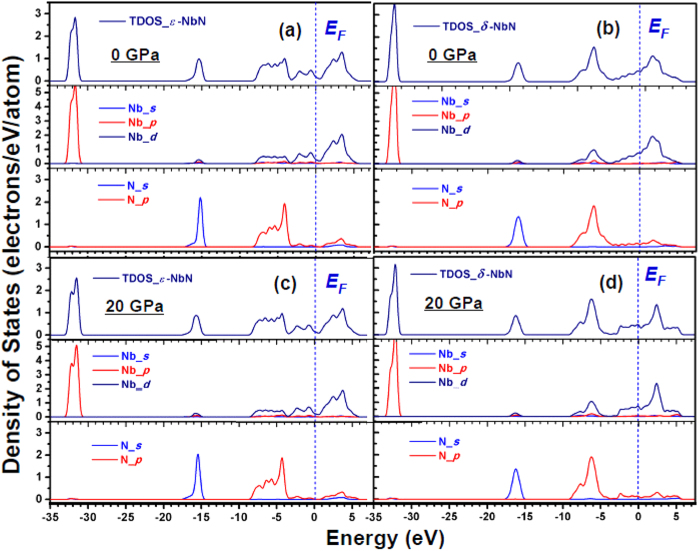
Total and partial density of states for hexagonal *ε*-NbN (**a**) and NaCl-structured *δ*-NbN (**b**) at 0 GPa, in comparison with those for hexagonal *ε*-NbN (**c**) and cubic *δ*-NbN (**d**) at a typical pressure of 20 GPa.

Theoretical calculations show that elastic and superconducting properties for transition-metal materials are closely related to their electronic properties^26^,^29^. Figures. 6a,c show strong hybridizations between Nb 4*d* and N 2*p* states in hexagonal *ε-*NbN with the appearance of a “pseudogap” just below or above the Fermi level (*E*_*F*_), suggesting the covalent and/or ionic bonding between Nb and N atoms. It is obvious that the TDOS for *ε*-NbN around the *E*_*F*_ lies in a dip, whereas the TDOS for *δ*-NbN increases monotonically at *E*_*F*_ (Figs. 6b,d). This indicates that the hexagonal NbN (*e.g.* WC-type-structure NbN, *ε*-NbN) was more stable than the cubic counterpart, agreeing with the total-energy calculations results^16^.

With increasing pressure from 0 to 20 GPa, the total DOS at the Fermi level *N*(*E*_*F*_) decreases from 0.176 to 0.163 electrons/eV atom, resulting in a decrease of the electron-electron interaction parameter μ*, which is strongly related to the superconducting transition temperature. To explore quantitatively the possible behavior of the superconducting properties of *ε*-NbN, we utilize the McMillan formula^33^, 
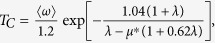
where μ* denotes the Coulomb pseudopotential, showing the repulsive interaction between electrons, *λ* is electron-phonon coupling constant, given by *λ* = *N(E*_*F*_*)<I*^2^>*/M<ω*^2^>, where <*I*^2^> is the average over the Fermi surface of square of the electronic matrix element for electron-phonon interaction, *M* is the atomic mass, and <*ω*^2^> is the square-averaged phonon frequency. The repulsive electron-electron interaction parameter μ* is calculated by applying the empirical relation^35^,^36^,^37^
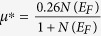
, where the total DOS at the Fermi level of *N*(*E*_*F*_) is expressed as unit of electrons/eV cell^−1^, and has the calculated value of 0.704 (Fig. 6a), yielding μ* ≈ 0.107. Taking our acoustic Debye temperature at ambient pressure *Θ*_*0*_ = 738 K, μ* ≈ 0.107, and the values of *λ* = 0.44~0.7 estimated from the theoretical values for *hcp-*structured MoC (*λ* = 0.44)^38^ and hexagonal *δ*-MoN (*λ* = 0.698)^36^, we obtain a predicted range for the superconducting critical temperature (*T*_*C*_) of 4.6~20.0 K, indicating that the hexagonal-structured *ε*-NbN may be a new superconductor and its transition temperature might be comparable to those for cubic *δ-*NbN (~17 K)^39^, *δ-*NbN_0.9_ (~12.4 K)^3^, and hexagonal WC-type *δ-*MoN (~14 K)^36^. In contrast, previous experimental studies on NbN_x_ films suggested that both of the tetragonal phases *γ*-Nb_4_N_3_ and Nb_4_N_5_ with long-range-ordered arrangement of vacancies exhibited superconducting properties, whereas the hexagonal NbN and Nb_5_N_6_ didn’t show superconductivity at temperatures down to 1.77 K (Ref. 40). The corresponding mechanisms for superconductivity in these transition-metal nitrides with different structures still need to be further investigated in future studies. It is suggested that the continuous promotion of *s*, *p* electrons to the *d* shell in all solids under pressure is one of the factors which will induce superconductivity^36^. As seen from Fig. 6, the contribution of the 4*d-*state is larger than those of the 5*s* and 5*p* states. The larger contribution of 4*d* state electrons clearly shows the possibility of superconductivity in hexagonal-structured NbN at ambient pressure.

To further verify the prediction of the superconducting properties of hexagonal *ε*-NbN, we calculated the superconducting critical temperature (*T*_*C*_) of cubic *δ-*NbN using the McMillan formula^35^,^36^,^37^. The value of the electron-electron interaction parameter μ* of *δ-*NbN at 0 GPa is determined from the empirical relation above to be ∼0.168 by using our value of the total DOS of *N*(*E*_*F*_) = 1.82 electrons/eV cell^−1^ at the Fermi level (Fig. 6b). By taking the theoretical Debye temperature^15^ *Θ*_*0*_ = 629 K for stoichiometric *δ-*NbN and the electron-phonon coupling constant *λ* = 0.87 for *δ-*NbN_0.9_ (Ref. 3), the superconducting critical temperature (*T*_*C*_) is predicted to be 18.3 K, which is in excellent agreement with the experimentally measured *T*_*C*_ = 17 K for *δ-*NbN film^41^, and the theoretically calculated result of *T*_*C*_ = 17.1 K^42^.

On the assumption that the superconducting properties of *ε*-NbN can be described by the McMillan equation^35^, we can speculate about the possible pressure dependence of *T*_*C*_ for this phase. It is noted that in the McMillan equation^35^ the critical temperature *T*_*C*_ strongly depends on the Debye temperature *Θ* (unit in K), but the dependence is quite complicated because it appears both in the linear and exponential term (from the <*ω*^2^> term in the expression of λ, as 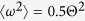
(Refs. 35, 43) To understand how the changes in Debye temperature affect the pressure dependence of *T*_*C*_, we assume the parameters <*I*^2^> and μ* to be constant with μ* = 0.107, and then make a numerical analysis. With the increase of pressure from 0 to 20 GPa, the Debye temperature (*Θ*), derived from the acoustic data, increases from 738 to 778 *K*, resulting in a positive factor up to ~1.05 in the linear term of McMillan formula. As for the exponential term, the enhancement of the *Θ* decreases λ to about 0.9λ, which in turn makes *T*_*C*_ fall to about 0.76*T*_*C*_ with the assumption of the initial values of *Θ =*738 K, μ* = 0.107 and λ = 0.7. Clearly, the change of *Θ* in the exponential term will be much more effective than in the linear term for the determination of *T*_*C*_, indicating that the increase of *Θ* plays a negative role in d*T*_*C*_/d*P*. However, the *N*(*E*_*F*_) decreases from 0.176 to 0.163 electrons/eV atom (or 0.704 to 0.652 electrons/eV cell^−1^) with increasing pressure from 0 to 20 GPa, as obtained by our first-principles calculations, resulting in the decrease of μ* and the subsequent enhancement of *T*_*C*_. If μ* is less pressure dependent, however, the changes of the electronic contribution in the pressure-induced electron-phonon interaction may yield a positive contribution to the increase of *T*_*C*_, which is consistent with the positive experimental value of d*T*_*C*_/d*P* for superconducting *δ*-NbN, as reported by Chen *et al.*^3^ The increase in *T*_*C*_ with pressure may be due to the continuous promotion of *s* to *d* electron transfer under high pressure.

In summary, the ultra-incompressibility, high shear rigidity and structural stability of *ε*-NbN have been measured at high pressure for the first time using ultrasonic interferometry and *in situ* X-ray diffraction techniques. Using a finite strain equation of state approach, the bulk and shear moduli, as well as their pressure derivatives, are derived from the measured velocities and densities, yielding *B*_*S0*_ = 373.3(15) GPa, *G*_*0*_ = 200.5(8) GPa, *∂B*_*S*_*/∂P* = 3.81(3), and ∂*G*/∂*P *= 1.67(1). Our obtained bulk modulus (*B*_*S0*_ = 373.3 GPa) is very close to that of *c*BN (381.1 GPa)^19^, and the shear modulus/rigidity (*G*_*0*_ = 200.5 GPa) is comparable to that for superhard *γ*-B (*G*_*0*_ = 227.2 GPa)^27^. Our calculated Vickers hardness of ~18.5 GPa is almost the same as that for *δ*-NbN^4^. The present Pugh modulus ratio *k* = *B/G* and Poisson’s ratio *v* increase with increasing pressure, and reaches *k* = 1.92 and *v* = 0.278 at 20 GPa, indicating that the *ε*-NbN becomes more ductile under high pressure. In addition, the pressure dependence of the Debye temperature (d*Θ/*d*P*), melting curve (*P*-*T*_*m*_), as well as the possible superconducting properties are also discussed. Based on our calculated Debye temperature (*Θ*), electron-electron interaction parameter (μ*), and the assumed value of the electron-phonon coupling constant (*λ*), by applying the McMillan formula^33^,^34^,^35^, a range of values of the superconducting temperature *T*_*C*_ of *ε*-NbN is predicted to be 4.6~20 K. The superconductivity in hexagonal structured *ε*-NbN may be related to its electronic properties as well as the structure itself.

## Methods

### High-Pressure Synthesis of Polycrystalline Hexagonal *ε-*NbN

The polycrystalline *ε-*NbN sample for the present ultrasonic measurement was hot-pressed at 10 GPa and 1100 °C for 1.5 hour in a multi-anvil apparatus at the High-Pressure Laboratory of Stony Brook University. Niobium nitride powder was used as starting material (Goodfellow, claimed 99% purity). Details of this experimental setup were described elsewhere^44^,^45^.

### Sound Velocity Measurements on *ε*-NbN at High Pressure

Elastic wave velocities of polycrystalline *ε*-NbN at high pressure were measured using ultrasonic interferometry technique in a multi-anvil apparatus. Details of this experimental setup for the ultrasonic measurements were described elsewhere^44^,^45^. The sample length under high pressure was determined by Cook’s method^21^ using the initial sample length, the zero-pressure density and the elastic wave velocity at high pressure. The length of the recovered sample was within ±1 *μ*m of the initial value, and virtually no plastic deformation of the sample was observed. Compressional and wave velocities of the sample at high pressure were calculated using the calculated sample length and the travel times. The bulk and shear moduli, as well as their pressure derivatives, were determined using Eulerian third-order finite strain equations^23^,^45^,^46^. The overall uncertainties in the present determination of the bulk and shear moduli are less than 1.5% of the nominal values. In this study, pressure was determined using the *P*-*t* relations 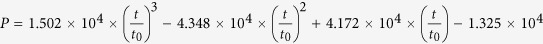, where *P* is cell pressure (in GPa), *t* is the S-wave travel time in the alumina buffer rod, and *t*_*0*_ is the S-wave travel time at room pressure (for further details of the use of alumina as a pressure marker, see Refs. 22, 46, 47).

### High-Pressure *In Situ* X-ray Diffraction Studies on Hexagonal *ε*-NbN

High-pressure synchrotron X-ray experiments using diamond-anvil cell (DAC) techniques were performed at the X17C beamline of National Synchrotron Light Source. The niobium nitride powders were loaded into the specimen hole in the gasket (stainless T301 steel) with methanol-ethanol (4:1) as pressure medium. The cell-pressure was determined from the fluorescence shift of ruby under high pressure^48^.

### First-Principles Calculations

Our first-principles calculations were performed with the CASTEP code^49^, based on density functional theory (DFT) using Vanderbilt-type ultrasoft pseudopotentials and a plane-wave expansion of the wave functions^50^. The local density approximation (LDA) was employed for determination of the exchange and correlation potentials for electron-electron interactions. The Broyden-Fletcher-Goldfarb-Shanno optimization method was applied to search for the ground states of hexagonal *ε*-NbN. For the Brillouin-zone sampling, the Monkhorst-Pack scheme^51^ was adopted. To confirm the convergence of our calculations, we have carefully analyzed the dependences of the total energy on the cutoff energy and the *k*-point set mesh according to the Monkhorst-Pack grid. During our first-principles calculations, the difference in total energy was minimized to below 5 × 10^−7^ eV/atom, the maximum ionic Hellmann-Feynman force is converged to less than 0.01 eV/Å, and the total stress tensor is reduced to the order of 0.02 GPa by using the finite basis-set corrections. The valance configuration is 4*p*^6^5*s*^1^4*d*^4^ and 2*s*^2^2*p*^3^ for Nb and N, respectively. Integrations in the Brillouin zone are performed using special k points generated with 10 × 10 × 2. One-electron valence states are expanded on a basis of plane waves with a cutoff energy of 600 eV in the electronic property calculations. All these parameters have been tested to be sufficient for the convergence.

## Additional Information

**How to cite this article**: Zou, Y. *et al.* Hexagonal-structured *ε*-NbN: ultra-incompressibility, high shear rigidity, and a possible hard superconducting material. *Sci. Rep.* **5**, 10811; doi: 10.1038/srep10811 (2015).

## Figures and Tables

**Table 1:** Physical properties of hexagonal *ε*-NbN at high pressure using *in situ* ultrasonic elastic wave velocity measurements.

*P**(GPa)**	***L*****(mm)**	***ρ*****(g/cm**^3^)	***V***_***P***_**(km/s)**	***V***_***S***_**(km/s)**	***B***_***S***_**(GPa)**	***G*****(GPa)**	***v***
***compression***							
2.6	1.586(2)	8.36(1)	8.86(1)	4.95(1)	383.4(18)	204.9(7)	0.2732
3.3	1.585(2)	8.38(1)	8.88(1)	4.96(1)	385.9(18)	206.0(7)	0.2734
4.1	1.584(2)	8.39(1)	8.90(1)	4.97(1)	388.8(18)	207.3(7)	0.2737
5.1	1.583(2)	8.41(1)	8.93(1)	4.98(1)	392.5(18)	208.9(7)	0.2740
6.6	1.581(2)	8.45(1)	8.97(1)	5.00(1)	398.2(19)	211.4(7)	0.2745
7.8	1.579(2)	8.47(1)	9.01(1)	5.02(1)	402.8(19)	213.4(7)	0.2749
8.9	1.578(2)	8.50(1)	9.04(1)	5.03(1)	406.9(19)	215.2(7)	0.2752
9.7	1.577(2)	8.51(1)	9.06(1)	5.04(1)	410.3(19)	216.7(7)	0.2755
10.0	1.576(2)	8.52(1)	9.07(1)	5.05(1)	411.4(19)	217.1(7)	0.2756
10.6	1.576(2)	8.53(1)	9.09(1)	5.06(1)	413.5(19)	218.1(7)	0.2757
10.9	1.575(2)	8.54(1)	9.09(1)	5.06(1)	414.6(19)	218.5(7)	0.2758
11.3	1.575(2)	8.55(1)	9.11(1)	5.07(1)	416.3(20)	219.3(8)	0.2760
11.7	1.574(2)	8.55(1)	9.12(1)	5.07(1)	417.7(20)	219.9(8)	0.2761
***decompression***							
8.3	1.579(2)	8.48(1)	9.02(1)	5.03(1)	404.7(19)	214.2(7)	0.2750
6.1	1.582(2)	8.44(1)	8.96(1)	5.00(1)	396.3(18)	210.6(7)	0.2743
5.3	1.583(2)	8.42(1)	8.94(1)	4.99(1)	393.3(18)	209.3(7)	0.2740
4.4	1.584(2)	8.40(1)	8.91(1)	4.97(1)	389.9(18)	207.8(7)	0.2737
2.6	1.586(2)	8.36(1)	8.86(1)	4.95(1)	383.4(18)	204.9(7)	0.2732
1.9	1.587(2)	8.34(1)	8.84(1)	4.94(1)	380.6(18)	203.7(7)	0.2729

^*^Pressures are determined from the relationship between the shear-wave travel times of Al_2_O_3_ buffer rod and the generated pressure determined by NaCl pressure scale using *in situ* X-ray diffraction^46^. The sample lengths at different pressures are calculated using the Cook’s method^19^. High-pressure densities are calculated using the equation *ρ* = *ρ*_*0*_ × (*L*_*0*_/*L*)^3^ and the initial bulk density (*ρ*_*0*_ = 8.30(2) g/cm^3^) at ambient condition, which is measured by Archimedes immersion method. The uncertainties are less than 0.3% in elastic wave shear velocities and less than 1.5% in the derived elastic moduli.

**Table 2:** Summary of single-crystal elastic constants, the Voigt bulk modulus (*B*), and shear modulus (*G*) for hexagonal *ε*-NbN and cubic *δ*-NbN obtained from our first-principles calculations, compared with those from the present sound velocity measurements and the previous studies on superhard *c*-BN (all in GPa)*.

**Materials**	***C***_***11***_	***C***_***33***_	***C***_***44***_	***C***_***12***_	***C***_***33***_	***B***	***G***	**Ref.**
*ε-*NbN (*hex*)	504	813	256	304	180	355	199	This study (theor.)
*ε-*NbN (*hex*)	—	—	—	—	—	373.2	200.5	This study (exper.)
*δ*-NbN (cubic)	739	—	76	161	—	354	161	Ref. 4
*c*-BN	786	—	445	172	—	381	400	Ref. 19

**Table 3:** Summary of the bulk modulus (*B*_*S*_), shear modulus (*G*), pressure dependences (*∂Bs/∂P, ∂G/∂P*), pugh modulus ratio (*k* *=* *B*_*S*_ */G*), poisson’s ratio (*v*) of the hexagonal *ε*-NbN, compared with those of the transition-metal nitrides and some typical superhard materials

**Materials**	***B***_***S0***_**(GPa)**	***G***_***0***_**(GPa)**	***∂Bs/∂P***	***∂G/∂P***	***B***_***S***_***/G***	*v*	**Ref.**
*ε-*NbN	373.2(1)	200.5(1)	3.81(3)	1.67(1)	1.861	0.272	This study
*δ*-NbN	354	161	4.36	—	2.199	0.303	Ref. 4
WC type-TaN	398	274	3.83	—	1.452	0.220	
WC type-NbN	357	226	3.89	—	1.579	0.238	Ref. 26
WC type-VN	336	220	3.82	—	1.527	0.230	
*δ*_3_*-*MoN (hex)	379.4	248.2	4.44	—	1.529	0.231	Ref. 28
*c*BN	381.1	398.8	—	—	0.956	0.112	Ref. 19
*γ*-B	213.9	227.2	—	—	0.941	0.108	Ref. 27
